# 2576. Piperacillin-Tazobactam or Cefepime for Treatment of Third Generation Cephalosporin Resistant Pneumonia in Intensive Care Unit Patients

**DOI:** 10.1093/ofid/ofad500.2192

**Published:** 2023-11-27

**Authors:** Clare DeLaurentis, Todd W Hokunson, Brian Nelson, Medini K Annavajhala, Anne-Catrin Uhlemann, Angela Gomez-Simmonds

**Affiliations:** Columbia University Medical Center, New York, New York; Columbia University Medical Center, New York, New York; NewYork-Presbyterian Hospital, New York, NY; Columbia University, New York, New York; Columbia University Irving Medical Center, New York, NY; Columbia University Irving Medical Center, New York, NY

## Abstract

**Background:**

Infections caused by Enterobacterales resistant to third generation cephalosporins (Ceph-RE) are rising in incidence. Carbapenems are frequently used to treat Ceph-RE infections based largely on studies of patients with bacteremia. Data evaluating alternative antibiotic options in pneumonia are limited. We assessed outcomes in intensive care unit (ICU) patients with Ceph-RE pneumonia treated with piperacillin-tazobactam (TZP) and cefepime (FEP) versus carbapenems.

**Methods:**

Adult ICU patients with pneumonia and positive respiratory culture for Ceph-RE between 1/2015-7/2022 were identified from electronic records. The antibiotic given after sensitivity test results and for > 72 hours (h) was termed the definitive agent. Patients who had polymicrobial pneumonia, severe infection at another body site, or were treated for < 72h or with an agent other than a carbapenem, TZP, or FEP were excluded. Nanopore sequencing was used to identify β-lactamase genes (n=68), and TZP and FEP minimum inhibitory concentrations (MIC) were determined by broth microdilution (n=64). The primary outcome was survival to 30 days or hospital discharge. Outcomes were compared in univariable and multivariable analyses.

**Results:**

Of 81 included patients, 56% were treated with a carbapenem and 44% received TZP or FEP. Patients receiving carbapenems had numerically higher Pitt Bacteremia Scores (PBS) and rates of severe sepsis (Figure 1). 52/68 isolates harbored an extended-spectrum β-lactamase gene; AmpCs were identified in 13 isolates and OXA-1 in 12. The majority of isolates were susceptible to TZP (91%) and FEP (64%). 73% and 92% of patients treated with TZP and FEP, respectively, met the primary outcome versus 67% of carbapenem recipients. In a multivariable model, only severe sepsis/septic shock was an independent predictor of poor outcome (OR 3.1, 95% CI 1.1-9.7, *p*=0.04); TZP or FEP use trended toward improved survival (OR 0.3, 95% CI 0.1-1.05, *p*=0.07). Survival did not differ based on the underlying resistance mechanism.

**Conclusion:**

While ICU patients receiving carbapenems had more severe illness and worse outcomes, TZP and FEP appeared to be effective agents for patients with less severe pneumonia. Alternative therapies are essential to curb ongoing increases in carbapenem resistance.

**Disclosures:**

**Anne-Catrin Uhlemann, MD, PhD**, Merck: Grant/Research Support

